# Moderate Dietary *Lactobacillus acidophilus* Supplementation Enhances Mid-Growth Nutrient Utilization and Shifts Intestinal *Lactobacillus* and *Escherichia coli* Counts in Yellow-Feathered Broilers

**DOI:** 10.3390/ani16121778

**Published:** 2026-06-09

**Authors:** Buheliqiemu Yushanaji, Xiao Zhang, Tian Tian, Qianqian Kou, Junmei Li, Jiancheng Liu, Fengming Li

**Affiliations:** 1 College of Animal Science, Xinjiang Agricultural University, Urumqi 830052, China; 18997898373@163.com (B.Y.); 18139230168@163.com (X.Z.); m15099660967_1@163.com (T.T.); 15348010072@163.com (J.L.); liujc@xjau.edu.cn (J.L.); 2 Xinjiang Branch of National Center for International Research on Animal Gut Nutrition, Xinjiang Agricultural University, Urumqi 830052, China; 3 Xinjiang Taikun Group Co., Ltd., Changji 831100, China; wd1615@126.com

**Keywords:** yellow-feathered broiler chickens, *L. acidophilus*, apparent nutrient digestibility, intestinal bacterial counts, intestinal morphology

## Abstract

This study tested the hypothesis that dietary *L. acidophilus* would improve nutrient utilization in yellow-feathered broilers mainly through intestinal changes rather than a consistent increase in growth performance. A total of 195 one-day-old broilers were assigned to a control group (basal diet), a T1 group (basal diet with 10 g/kg *L. acidophilus*), or a T2 group (basal diet with 15 g/kg *L. acidophilus*), with five replicates of 13 birds per treatment over 63 days. The biological responses followed a clear pattern. First, supplementation did not significantly affect average daily feed intake, average daily gain, or feed-to-gain ratio at any growth stage or across the entire trial (days 1–63; *p* > 0.05), indicating limited direct effects on growth. Second, the main nutritional response occurred during days 22–42, when T1 increased the apparent nutrient digestibility of dry matter, organic matter, ether extract, crude protein, and gross energy compared to the control (*p* < 0.05). Third, intestinal responses aligned with localized gut modulation: both supplemented groups had higher culture-based *Lactobacillus* counts and lower *E. coli* counts in the cecum and ileum (*p* < 0.05), and T1 increased duodenal villus height (*p* < 0.05). In contrast, serum biochemical, immune, and antioxidant indices showed only isolated, phase-dependent changes and were not considered the primary response. Overall, moderate dietary supplementation with *L. acidophilus* enhanced nutrient utilization during mid-growth and was linked to targeted intestinal bacterial and morphological changes, but it did not consistently improve growth performance in yellow-feathered broilers.

## Simple Summary

Reducing antibiotic use while maintaining poultry health and efficient nutrient absorption is a key goal in poultry production. Probiotics such as *Lactobacillus acidophilus* are often viewed as potential alternatives, but their effects on growth performance do not always improve it. In this study, we examined whether dietary supplementation with *L. acidophilus* could enhance overall growth or mainly influence nutrient utilization and gut health in yellow-feathered broilers. The results showed that supplementation did not significantly improve average daily feed intake, average daily gain, or feed conversion throughout the entire trial. However, the moderate inclusion level (10 g/kg) increased the apparent nutrient digestibility of several nutrients during the mid-growth phase, and both supplemented groups had higher *Lactobacillus* counts and lower *Escherichia coli* counts in the cecum and ileum. Additionally, duodenal villus height was higher in the 10 g/kg group. These findings suggest that *L. acidophilus* has limited effects on overall production performance but may support gut function and nutrient absorption at specific growth stages in yellow-feathered broilers.

## 1. Introduction

In the current poultry industry, which faces the dual challenges of efficient production and environmental safety, probiotics—particularly *L. acidophilus*—have become essential for healthy poultry farming because they improve gut health and reduce reliance on antibiotics [1]. This shift supports global efforts to reduce antibiotic use, as overuse has led to increased resistance and antibiotic residues in meat products [2]. Probiotics function through several mechanisms, including regulating gut microbiota composition, strengthening the intestinal barrier, and modulating immune and metabolic functions. These actions lower pathogen levels and enhance nutrient absorption [3,4]. *L. acidophilus*, a common probiotic lactic acid bacterium, exhibits traits such as acid resistance, acid production, and competitive exclusion. These qualities enable it to colonize the intestine and prevent potential pathogens [5]. For example, probiotics inhibit pathogen colonization and growth by competing for limited space and nutrients within the gut [6]. Additionally, the metabolic products of *L. acidophilus* are key to its probiotic effects. It ferments sugars into lactic acid, which acts as a postbiotic, significantly lowering gut pH and creating an environment hostile to many pathogens, including Salmonella [7,8]. Furthermore, *L. acidophilus* interacts with the host’s intestinal environment through its metabolic activities, helping to maintain the integrity of the intestinal barrier—the body’s primary defense against pathogens and toxins [9]. Studies in mouse models demonstrate that *L. acidophilus* can resist Salmonella typhimurium infection and exert beneficial effects by regulating the gut microbiome, transcriptome, and metabolites [9].

In poultry production, the biological effects of probiotics are influenced not only by their inclusion in the diet but also by interactions among strain traits, supplementation levels, host genotype, and the production environment [10]. Even within the *Lactobacillus* genus, probiotic strains can differ significantly in their ability to colonize, competitively exclude harmful bacteria, and affect intestinal barrier integrity and host inflammatory responses [4,11]. Consequently, probiotic supplementation does not always produce consistent improvements in growth performance, and positive effects on the intestine may occur without noticeable changes in body weight gain or feed efficiency [12]. This issue is also relevant in yellow-feathered broilers, where previous probiotic studies have shown alterations in gut microbiota, intestinal structure, or feed efficiency, but not always broad or uniform gains in production traits [13].

Among the candidate probiotics used in poultry, *L. acidophilus* is particularly interesting because it may regulate intestinal physiology through several interconnected mechanisms, including acidifying the gut environment, inhibiting potential pathogens via competitive exclusion, supporting epithelial barrier function, and modulating immune and inflammation-related responses [3,10]. However, whether these intestinal-level effects are sufficient to improve nutrient utilization and production outcomes in yellow-feathered broilers remains unclear, and the response may depend on the level and form of probiotic supplementation [11]. More specifically, the response to *L. acidophilus* can be understood through a sequential intestinal metabolic model rather than a direct growth-promotion model. At the intestinal level, strain-dependent colonization ability and competitive exclusion may change the relative amounts or culturable counts of beneficial and potentially harmful bacteria. These microbial shifts may then affect fermentation-related metabolites, including organic acids, which can help lower intestinal pH, inhibit enteric pathogens, and regulate mucosal immune tone. Meanwhile, probiotic-driven changes in microbial stimulation and local metabolites may influence epithelial renewal, villus growth, and barrier function, thereby altering the absorptive surface area available for nutrient digestion and absorption. From this perspective, changes in apparent nutrient metabolic rates are expected to occur after intestinal bacterial and morphological responses, while improvements in growth performance depend on these intestinal effects being strong, persistent, and aligned with the host’s growth stage. Therefore, it is important to evaluate both moderate and high inclusion levels, since probiotic responses might be nonlinear. A moderate dose may promote beneficial intestinal regulation, whereas a higher dose might not provide additional benefits due to ecological niche saturation, substrate competition, or host adaptation.

Therefore, this study aimed to evaluate the effects of two dietary inclusion levels of *L. acidophilus* on growth performance, apparent nutrient digestibility, serum physiological indices, intestinal bacterial counts, and intestinal morphology in yellow-feathered broilers. We hypothesized that dietary *L. acidophilus* would primarily influence nutrient utilization and intestinal traits, rather than necessarily resulting in a consistent increase in overall growth performance.

## 2. Materials and Methods

### 2.1. Growth Performance Recording and Calculation

A total of 195 one-day-old yellow-feathered broiler chicks with an initial body weight of 25.03 ± 0.74 g were used in this experiment. Birds were assigned to three dietary treatments with five replicate pens per treatment and 13 birds per pen. The control group received the basal corn–soybean meal diet. The two supplemented groups received the same basal diet with *Lactobacillus acidophilus* added at 10 g/kg (T1) or 15 g/kg (T2). The *L. acidophilus* preparation was obtained from Xi’an Wanfang Biotechnology Co., Ltd. (Xi’an, China), containing at least 1.0 × 10^10 CFU/g of viable bacteria. The experiment lasted 63 days. The basal diet composition was the same as in our previous study on raffinose supplementation in yellow-feathered broilers, as both experiments were conducted under similar production conditions and used the same basal control diet [14]. This study differed from the previous work in the dietary additive evaluated and in the biological focus, which centered on probiotic-associated nutrient utilization and intestinal responses. The ingredients and nutrient levels of the basal diets are detailed in Table 1.

The chicks were fed pelleted diets from day 1 to day 63, as shown in Table 1. These birds were raised in floor pens following standard commercial management practices. Feed and water were available free choice throughout the experiment. Temperature and lighting schedules were maintained according to the routine requirements for yellow-feathered broilers of that age. All birds were kept under identical environmental and sanitary conditions throughout the trial. Birds were reared in floor pens with clean, dry litter under the same poultry-house conditions throughout the trial. Feed and water were provided ad libitum. The room temperature was maintained at approximately 33–35 °C during the first week and then gradually decreased by 2–3 °C per week until it reached about 24 °C. A near-continuous lighting schedule was used during the first week, followed by a standard light cycle for yellow-feathered broilers during later growth stages. Ventilation was adjusted daily to maintain air quality and reduce moisture buildup. Pens, drinkers, and feeders were cleaned regularly, and birds were monitored daily for health and mortality.

Samples of each experimental diet were collected from every feeding stage for nutrient analysis. Dry matter was measured by oven drying in accordance with GB/T 6435-2014 [15], and organic matter was calculated after ash determination in accordance with GB/T 6438-2007 [16]. Crude protein was analyzed using GB/T 6432-2018 [17]. The values for metabolizable energy, calcium, total phosphorus, and lysine listed in Table 1 were estimated based on the feed formulation and ingredient composition database rather than directly measured.

### 2.2. Measurement of Growth Performance

Growth performance was summarized at the replicate-pen level for each growth phase (days 1–21, 22–42, 43–63, and 1–63). Birds were weighed by pen at the start and end of each phase, feed was provided, and refusals were recorded for the same periods. Average daily feed intake (ADFI), average daily gain (ADG), and feed conversion ratio (FCR) were calculated from replicate-pen records rather than from treatment-level means. In these calculations, bird-days refer to the number of birds in a replicate pen multiplied by the number of feeding days in the respective phase; when mortality occurred, bird-days were adjusted accordingly. The indices were calculated as follows:

$$ADFI=\frac{Feed\;offered-Feed\;refusal}{Number\;of\;bird-days}$$

$$ADG=\frac{Final\;body\;weight-Initial\;body\;weight}{Number\;of\;bird-days}$$

$$F:G=\frac{ADFI}{ADG}$$

### 2.3. Carcass Trait Evaluation

On day 63, two birds from each replicate pen were selected for carcass evaluation after a 12-h feed withdrawal, with free access to water. Each bird was weighed immediately prior to slaughter to determine the pre-slaughter live body weight. After exsanguination and defeathering, the dressed carcass was weighed and used to calculate the dressing percentage. The semi-eviscerated carcass weight was recorded after removing the trachea, esophagus, crop, intestines, spleen, pancreas, gallbladder, and reproductive organs. The eviscerated carcass weight was then measured following the removal of the head, feet, abdominal fat, and remaining internal organs. Breast and thigh muscles from both sides of the carcass were dissected and weighed. Carcass traits were calculated using commonly used carcass-yield indices as follows:

Dressing percentage=Dressed Carcass weight/Pre-slaughter live body weight×100

Semi-eviscerated carcass yiled=Semi-eviscerated carcass weight/Pre-slaughter live body weight×100

Eviscerated carcass yield=Eviscerated carcass weight/Pre-slaughter live body weight×100

Breast muscle yield=Breast musle weight/Eviscerated carcass weight×100

Thigh muscle yield=Thigh muscle weight/Eviscerated carcass weight×100

### 2.4. Apparent Nutrient Digestibility

Apparent nutrient digestibility or retention was measured during the last four days of each experimental phase using the total excreta collection method at the replicate-pen level. Before collection, each pen was cleaned, and clean plastic sheets or collection trays were placed under the birds to reduce contamination from litter. Feed was offered, and refusals were weighed for each replicate during the collection period. Excreta were collected twice daily from each replicate pen. Feathers, spilled feed, and visible foreign material were manually removed. Daily excreta from the same replicate were pooled, weighed, mixed thoroughly, and stored at −20 °C. At the end of each collection period, pooled excreta samples were thawed, homogenized, and subsampled; approximately 100 g of each representative sample was dried at 65 °C for 48 h, ground, and stored at −20 °C until analysis. Dry matter (DM) and organic matter (OM) contents in diets and excreta were determined according to GB/T 6435-2014 and GB/T 6438-2007, respectively. Nitrogen was analyzed using the Kjeldahl method according to GB/T 6432-2018, and crude protein (CP) was calculated as nitrogen times 6.25. Gross energy (GE) was measured using bomb calorimetry. Apparent nutrient digestibility or retention was calculated as follows:

Apparentnutrientdigestibility=[(nutrient intake−nutrient excretion)/nutrient intake]×100

### 2.5. Serum Biochemical, Immune, and Antioxidant Measurements

Blood samples were collected on days 21, 42, and 63. At each sampling point, one bird from each replicate pen was randomly chosen (*n* = 5 per treatment). About 2 mL of jugular venous blood was drawn into sterile, non-anticoagulant tubes. Serum was separated after centrifugation at 3500 rpm for 10 min and stored at −20 °C before analysis. Serum biochemical indices, including total protein (TP), albumin (ALB), triglycerides (TG), total cholesterol (TCH), low-density lipoprotein (LDL), high-density lipoprotein (HDL), creatinine, uric acid, alanine aminotransferase (ALT), and aspartate aminotransferase (AST), were measured using commercial diagnostic kits following the manufacturers’ instructions and the methods described by Friedman and Young [10]. Serum immunoglobulin A (IgA), immunoglobulin G (IgG), and immunoglobulin M (IgM) were measured using commercial kits from Nanjing Jiancheng Bioengineering Institute (Nanjing, China).

Oxidative markers were assessed to determine how dietary additions affect antioxidant status. The total oxidant capacity (T-AOC) was measured using a spectrophotometer (Hitachi, Tokyo, Japan) and a commercial kit (Randox Labs, Crumlin, UK), as outlined by Erel [11]. The functions of glutathione peroxidase (GSH-Px), superoxide dismutase (SOD), and catalase (CAT) were evaluated following Wang et al. [12]. Malondialdehyde (MDA) was measured according to the methods established by Wang et al. [12] and Miranda et al. [13].

### 2.6. Volatile Fatty Acids

Cecal volatile fatty acids (VFAs) were measured using gas chromatography with 4-methylpentanoic acid as the internal standard, following a slightly modified version of the method described by Xu [18]. A mixed standard stock solution was prepared in a 100 mL volumetric flask by adding 300 μL of acetic acid, 130 μL of propionic acid, 70 μL of n-butyric acid, 12 μL of isobutyric acid, 15 μL of valeric acid, and 15 μL of isovaleric acid and then diluted to volume with ultrapure water. The final stock concentrations were 52.41 mmol/L acetic acid, 17.43 mmol/L propionic acid, 7.62 mmol/L butyric acid, 1.29 mmol/L isobutyric acid, 1.38 mmol/L valeric acid, and 1.36 mmol/L isovaleric acid.

A series of working standards was prepared by diluting 1.0, 0.8, 0.6, 0.4, 0.2, or 0.1 mL of the mixed stock solution with distilled water to a final volume of 1.0 mL. These standards were used to generate calibration curves for VFA quantification.

For sample preparation, cecal digesta was thawed at 4 °C and homogenized. About 0.4 g of digesta was transferred into a centrifuge tube, mixed with 2.5 volumes of double-distilled water, vortexed for 5 min, and centrifuged at 18,000× *g* for 30 min. Then, 0.5 mL of supernatant was mixed with 0.5 mL of 10% trichloroacetic acid and 0.1 mL of 40 mmol/L 4-methylpentanoic acid. After vortexing and standing for 20 min, the mixture was centrifuged again at 18,000× *g* for 30 min. The supernatant was transferred to an autosampler vial, and 0.5 μL was injected for gas chromatographic analysis. VFA concentrations were calculated using the internal-standard calibration curve.

The 10% trichloroacetic acid solution was prepared by dissolving 10 g of trichloroacetic acid in distilled water and adjusting the final volume to 100 mL. The 40 mmol/L 4-methylpentanoic acid solution was prepared by diluting 50.2 μL of 4-methylpentanoic acid to 10 mL with distilled water.

### 2.7. Culture-Based Enumeration of Intestinal Lactobacillus and E. coli

The cecal and ileal contents were collected for culture-based enumeration of *Lactobacillus* and *E. coli*. Briefly, 1 g of cecal or ileal content was homogenized in 99 mL of sterile saline, and the suspension was serially diluted 10-fold. Aliquots (0.3 mL) of appropriate dilutions were spread onto selective media. *Lactobacillus* was cultured on Lactobacilli de Man, Rogosa, and Sharpe (LBS) agar, while *E. coli* was cultured on Eosin Methylene Blue (EMB) agar. The inoculated plates were incubated at 37 °C for 24–48 h, after which colonies were counted. The results are expressed as log colony-forming units per gram of intestinal content (lg CFU/g). This culture-based method was designed to quantify culturable *Lactobacillus* and *E. coli* as targeted bacterial indicators. It was not intended to provide a comprehensive characterization of the intestinal microbiota. Therefore, the microbiological results should be interpreted as changes in selected culturable bacterial groups rather than as evidence of broad microbial community restructuring.

### 2.8. Histological Assessment of Intestinal Morphology

After blood collection and euthanasia, approximately 2 cm segments of the duodenum, jejunum, and ileum were excised, gently rinsed with physiological saline, and fixed in 4% paraformaldehyde for 24 h. The fixed tissues were dehydrated through graded ethanol, cleared in xylene, embedded in paraffin, and sectioned at 5 μm. Sections were stained with hematoxylin and eosin, mounted with neutral resin, and examined under a light microscope. Villus height (VH) and crypt depth (CD) were measured using NIS-Elements D imaging software (Nikon Instruments Inc., Melville, NY, USA; available at: https://www.microscope.healthcare.nikon.com/products/software/nis-elements, accessed on 5 June 2026). according to previously described intestinal histomorphometric methods in broilers [19]. VH was defined as the distance from the villus tip to the villus-crypt junction, while CD was measured from the crypt opening to the crypt base. The villus height-to-crypt depth ratio (V/C) was then calculated.

### 2.9. Statistical Analysis

All data were analyzed using the general linear model procedure of SPSS 26.0 (SPSS Inc., Chicago, IL, USA). Dietary treatment was considered the fixed effect. For growth performance and apparent nutrient digestibility, the replicate pen was used as the experimental unit (*n* = 5). For carcass traits, serum biochemical, immune, and antioxidant indices, cecal volatile fatty acids, intestinal bacterial counts, and intestinal morphology, one bird (or one sample) from each replicate was used as the experimental unit unless otherwise stated (*n* = 5 per treatment). When the overall treatment effect was significant, means were separated using Duncan’s multiple range test. In addition, orthogonal polynomial contrasts were used to evaluate linear and quadratic responses to increasing levels of dietary *L. acidophilus* supplementation. The statistical model used was as follows:

Y_ij_ = *μ* + T_i_ + e_ij_

where Y_ij_ represents observation, *μ* is the observed mean, T_i_ is the effect of treatments, and e_ij_ is the experimental random error.

Because only two supplementation levels of *L. acidophilus* were evaluated, along with the control, linear and quadratic contrasts were used to describe the response pattern across the tested inclusion levels rather than to establish a complete dose–response curve or determine an optimal dose. Additionally, given the relatively large number of physiological, metabolic, microbial, and morphological endpoints, the results should be interpreted hierarchically based on the biological hypothesis. Growth performance and apparent nutrient metabolic rate were considered the primary functional outcomes, while intestinal bacterial counts and intestinal morphology served as supportive endpoints. Serum biochemical, immune, antioxidant, and volatile fatty acid indices were regarded as secondary exploratory outcomes. No formal experiment-wise correction for multiple testing was applied across all endpoints; therefore, isolated significant responses, especially among secondary endpoints, should be interpreted cautiously.

For the variables listed in Tables 3–6, data were analyzed separately for each sampling period or growth phase. Sampling time was not included as an independent fixed effect in the primary model because each sampling time corresponded to a different age and dietary phase. Therefore, the *p*-values reported in Tables 3–6 reflect treatment, linear, and quadratic effects within each sampling period, rather than the main effect of sampling time or the treatment × sampling time interaction.

## 3. Results

### 3.1. Growth Efficiency

As shown in Table 2, dietary *L. acidophilus* supplementation did not significantly affect ADFI, ADG, or F:G during days 1–21, 22–42, 43–63, or over the entire 1–63 day period (*p* > 0.05). After verifying the data, ADFI during days 1–21 and days 1–63 was recalculated based on the respective feeding periods. The overall F for days 1–63 was also adjusted from total feed intake and total body-weight gain. Linear and quadratic responses were generally not significant for most growth traits (*p* > 0.05), although ADG showed a tendency to increase linearly during days 43–63 (*p* = 0.068). Overall, these findings indicate that dietary *L. acidophilus* supplementation did not consistently improve growth performance in yellow-feathered broilers.

### 3.2. Apparent Nutrient Digestibility

As shown in Table 3, dietary *L. acidophilus* supplementation affected apparent nutrient digestibility only during days 22–42. Significant treatment effects were detected for DM, OM, EE, CP, and GE (T column, *p* < 0.05). For DM, OM, EE, and CP, the T1 group had higher values than both the control and T2 groups, indicating a quadratic response, with the moderate supplementation level producing the greatest effect. For GE, both T1 and T2 were higher than those of the control group.

### 3.3. Blood Indicators

Table 4 shows that *L. acidophilus* had no significant overall effect on serum TP, ALB, GLB, and the albumin/globulin ratio (A/G) at various stages (*p* > 0.05), and no obvious changes were observed in TBIL (*p* > 0.05). During days 1–21, BUN was significantly affected by the treatment (*p* = 0.023) and increased linearly with the level of addition (*p* = 0.007), with both T1 and T2 higher than the control group; during days 22–42, there were no significant differences in any of the indicators (*p* > 0.05). In the 43–63-day stage, BUN again showed a significant treatment effect (*p* = 0.026) and a secondary effect (*p* = 0.010), characterized by an increase in T1 and a return of T2 to levels close to the control.

Table 5 shows that serum TG was affected by *L. acidophilus* supplementation during 1–21 d (*p* = 0.003), with T1 higher than the control and T2 intermediate (0.78 mmol/L). During 22–42 d, only AST differed among treatments (*p* = 0.037), with the lowest value in T2 (219.20 U/L) compared with the control (249.00 U/L) and T1 (239.60 U/L). No treatment effects were detected for the remaining indices within each stage (*p* > 0.05), although TG showed a tendency to vary during 43–63 d (*p* = 0.086).

### 3.4. Immunity and Antioxidative Properties

Table 6 shows that during 22–42 d, serum IgA, IgG, and IgM were not affected by *L. acidophilus* supplementation (*p* > 0.05). In contrast, antioxidant enzymes responded at this stage: CAT differed among treatments (*p* = 0.031), with higher activity in T2 than in the control, whereas GSH-Px also showed a treatment effect (*p* = 0.032), with the highest activity in T2 and the lowest in T1. T-AOC, SOD, and MDA were unchanged (*p* > 0.05). During 43–63 days, IgA decreased in the supplemented groups (*p* = 0.0279), whereas CAT increased markedly (*p* < 0.001), with a higher value at T1 (0.31 U/mL) than at T2 (0.22 U/mL) and the control (0.17 U/mL). SOD was lower in T1 and T2 than in the control (*p* = 0.039). No differences were detected for IgG, IgM, GSH-Px, T-AOC, or MDA at this stage (*p* > 0.05).

### 3.5. Slaughter Traits

As shown in Table 7, dietary supplementation with *L. acidophilus* had limited effects on the carcass traits of yellow-feathered broilers. Dressing percentage and breast muscle yield were not significantly affected by the treatment (*p* > 0.05). Semi-eviscerated carcass yield was lower in the T2 group than in the control group (*p* = 0.048), while eviscerated carcass yield only showed a tendency to differ among treatments (*p* = 0.077). Thigh muscle yield also tended to differ among treatments (*p* = 0.066), but the overall treatment effect was not significant. These results suggest that dietary *L. acidophilus* supplementation did not consistently improve carcass yield traits.

### 3.6. Volatile Fatty Acids

Table 8 indicates that varying amounts of *L. acidophilus* do not significantly impact the cecal VFAs in poultry (*p* > 0.05). However, in both experimental groups, VFA concentrations in the cecum were higher than in the control group.

### 3.7. Culture-Based Counts of Lactobacillus and E. coli

**Figure 1:**
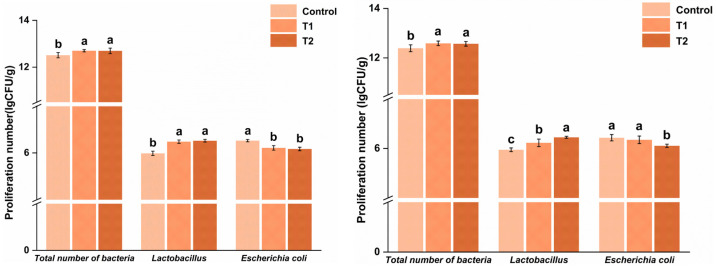
Effects of dietary *L. acidophilus* supplementation on culture-based counts of *Lactobacillus* and *E. coli* in the cecum and ileum of yellow-feathered broilers: T1, 10 g/kg *L. acidophilus*; T2, 15 g/kg *L. acidophilus*. (**Left**): Effect of *L. acidophilus* supplementation on the cecal bacterial count of yellow-feathered broilers; (**Right**): Effect of *L. acidophilus* supplementation on the number of ileal bacteria in yellow-feathered broilers. Different lowercase letters above the bars indicate significant differences among treatment groups within the same bacterial category and sampling time (*p* < 0.05), whereas bars sharing the same letter are not significantly different. Values are presented as means ± SEM.

As shown in Figure 1, dietary *L. acidophilus* supplementation influenced the culture-based counts of *Lactobacillus* and *E. coli* in both the cecum and ileum. In the cecum, *Lactobacillus* counts were significantly higher in the T1 and T2 groups compared to the control group, increasing by 8.36% and 9.03%, respectively (*p* < 0.05), while *E. coli* counts were significantly lower, decreasing by 4.98% and 5.83%, respectively (*p* < 0.05). Similar changes were seen in the ileum, where *Lactobacillus* counts increased by 4.70% and 8.40% in the T1 and T2 groups, respectively, whereas *E. coli* counts decreased by 1.42% and 5.40% compared to the control group (*p* < 0.05).

### 3.8. Intestinal Morphology

Table 9 shows that duodenal villus height was significantly higher in the T1 group than in the control group (*p* < 0.05), with a 34.27% increase. In contrast, there were no significant effects of treatment on duodenal crypt depth or the villus height/crypt depth ratio (*p* > 0.05). Similarly, no significant differences in jejunal or ileal morphology were observed among the treatments (*p* > 0.05).

## 4. Discussion

This study demonstrated that dietary *L. acidophilus* supplementation did not significantly influence ADFI, ADG, or F:G during any growth phase or throughout the entire experimental period. However, stage-specific responses were observed in apparent nutrient digestibility and certain intestinal-related parameters. Notably, the treatment was linked to higher cultured *Lactobacillus* counts, lower cultured *E. coli* counts, and increased duodenal villus height in the T1 group. These results suggest that *L. acidophilus* primarily supports intestinal regulation and nutrient utilization rather than directly promoting growth.

Probiotics are live microorganisms that provide benefits to the host by improving the balance of intestinal microbiota. They include various types such as bacteria, fungi, and yeasts. In poultry, common probiotic genera include Lactobacillus, Bifidobacterium, Bacillus, Streptococcus, and Saccharomyces [20]. These probiotics primarily enhance nutrient utilization and digestive efficiency by improving the intestinal environment [21]. Recent research indicates that supplementing poultry diets with probiotics can boost production performance [4,22]. *L. acidophilus*, as a probiotic, shows broad potential for use in poultry farming, especially in enhancing production, maintaining intestinal health, supporting immune function, increasing stress resistance, and providing an alternative to antibiotics [2]. In this study, after adding *L. acidophilus*, yellow-feathered broilers showed numerical changes in average daily feed intake (ADFI), average daily gain (ADG), and feed-to-gain ratio across different stages; however, most differences were not statistically significant. This may relate to the longer growth cycle of yellow-feathered broilers and their fat deposition and energy allocation patterns, which differ from those of white-feathered fast-growing broilers [23]. The role of probiotics in this research seemed to focus more on intestinal regulation and nutrient utilization than on consistent improvement in growth performance [24]. This aligns with the apparent nutrient digestibility results, which showed higher values for the T 1 group during 22–42 days, with no significant differences during 1–21 days or 43–63 days. This pattern suggests that the moderate inclusion level elicited a more pronounced response during the middle growth phase but did not produce sustained effects throughout the cycle [25]. In summary, dietary *L. acidophilus* caused numerical changes in ADFI, ADG, and F:G across growth stages; however, most of these differences were not statistically significant. This may be due to the longer growth cycle and distinct energy distribution pattern of yellow-feathered broilers compared to fast-growing white-feathered ones. The findings imply that the response to *L. acidophilus* did not consistently improve growth performance. Instead, the T 1 group demonstrated a more noticeable increase in apparent nutrient digestibility between 22 and 42 days, whereas the T 2 group did not show further gains compared to T 1. Under these conditions, this pattern suggests that a moderate inclusion level elicited a more reliable response than a higher one.

Slaughter rate, semi-eviscerated yield, and eviscerated yield, as well as breast muscle rate and leg muscle rate, reflect how nutrients are allocated in poultry and the efficiency of meat production. These are key endpoints for evaluating whether additives can direct more nutrients toward edible muscle tissue [26]. Studies have shown that adding probiotics to broiler diets does not significantly affect carcass performance [27,28]. In this study, *L. acidophilus* did not affect slaughter yield or pectoral muscle yield (*p* > 0.05), whereas the full-eviscerated yield differed among groups (*p* = 0.048), with T2 yielding a lower full-eviscerated yield than the control. The semi-eviscerated yield and leg muscle yield only showed tendencies to differ (*p* > 0.05). This suggests that when ADFI, ADG, and F/G show no significant changes over the entire period, it may be difficult to find broad, significant differences in slaughter rate and main carcass performance. In the present study, growth performance was not significantly affected at any growth phase or across the entire trial, indicating that dietary *L. acidophilus* did not substantially affect overall body weight gain. Under these conditions, major improvements in carcass traits would not be expected. The reduction in eviscerated yield observed in the T2 group, along with only trend-level changes in semi-eviscerated yield and thigh muscle yield, suggests a limited and isolated effect on carcass component distribution rather than consistent improvement in productive performance.

Serum biochemical indices are vital for assessing the physiological status of animals, especially regarding protein nutritional health, liver function, nitrogen metabolism, and lipid and carbohydrate balance [29]. Among these, TP, ALB, GLB, and A/G are commonly used to evaluate protein nutritional status and liver synthetic activity. BUN serves as a marker for nitrogen metabolism, although in poultry, nitrogen is mainly excreted as uric acid, and serum urea nitrogen levels are typically low and influenced by dietary protein intake, amino acid utilization, and intestinal nitrogen flow [30]. TBIL, ALT, and AST are key indicators of hepatobiliary health and hepatocellular integrity, while TG, TCH, HDL, LDL, and GLU reflect lipid and carbohydrate metabolism [31,32,33]. In this study, TP, ALB, GLB, A/G, and TBIL showed no significant changes at any stage, indicating that dietary *L. acidophilus* did not notably affect serum protein profiles. BUN levels increased significantly during days 1–21 (*p* = 0.023), and again during days 43–63 (*p* = 0.026), with higher levels observed in the 10 g/kg group and a return to control levels in the 15 g/kg group, suggesting stage-dependent regulation of nitrogen metabolism. ALT remained unchanged throughout. AST levels significantly decreased from days 22 to 42 (*p* = 0.037), especially in the 15 g/kg group, indicating a stage-specific shift in liver metabolic status during mid-growth. Concerning carbohydrate and lipid metabolism, GLU, HDL, LDL, and TCH were not significantly affected by *L. acidophilus* supplementation. Conversely, the temporary increase in TG during early growth did not persist, implying that the lipid response to supplementation was short-term rather than sustained. Overall, these findings suggest that *L. acidophilus*’s effect on serum biochemical metabolism was limited and stage-dependent, with the most notable effects observed in BUN, AST, and early-stage TG. However, since these responses were confined to specific phases and did not consistently impact growth performance or other major biochemical markers, their biological significance should be interpreted with caution.

Serum immunoglobulins (IgA, IgG, and IgM) are important biomarkers of humoral immunity. In poultry, especially chickens, secretory IgA (sIgA) more commonly functions in mucosal immunity [34]. Its dynamic changes are closely linked to the strength of intestinal mucosal antigen stimulation, barrier function, and interactions with the microbial community [35]. In this experiment, during the 22–42-day period, *L. acidophilus* had no significant effect on serum IgA, IgG, or IgM, and no noticeable systemic humoral immune enhancement or suppression was observed in the middle stage. From 43 to 63 days, serum IgA in the experimental group was significantly lower than in the control group, while IgG and IgM showed no significant differences (*p* > 0.05). This change may occur because, when the intestinal condition remains fairly stable, *L. acidophilus* can reduce antigenic stimulation by adjusting certain gut-related factors, including the balance between the two bacterial groups studied here. In such cases, the body might no longer need to produce as much IgA, leading to a decrease in serum IgA levels or a return to normal balance [36]. Notably, the antioxidant responses during 43–63 days were not consistent because CAT increased while SOD decreased in the supplemented groups, whereas T-AOC, GSH-Px, and MDA remained unchanged. This pattern shows that dietary *L. acidophilus* did not uniformly enhance systemic antioxidant status but instead elicited selective, phase-dependent changes in specific antioxidant enzymes. The inconsistent responses of CAT and SOD indicate that dietary *L. acidophilus* did not consistently improve systemic antioxidant capacity. Because intestinal oxidative status, inflammatory signaling, and antioxidant-related gene expression were not measured, these enzyme changes should be seen as phase-dependent physiological responses rather than as a specific mechanistic pathway.

Volatile fatty acids, primarily consisting of acetic acid, propionic acid, and butyric acid, are the main metabolic byproducts produced by anaerobic fermentation of indigestible carbohydrates by intestinal microorganisms [28]. In poultry, they act as a key indicator of gut microbiota function. Although the cecum in poultry contributes minimally to energy metabolism directly, VFAs play crucial roles in regulating gut pH, inhibiting pathogenic bacteria such as Salmonella, supporting energy metabolism in intestinal epithelial cells, strengthening barrier function, and maintaining immune homeostasis [37,38]. Studies show that under conditions of higher baseline nutrient metabolism and stable rearing environments, probiotics tend to optimize VFA composition—particularly increasing the proportion of butyric acid—rather than greatly increasing total VFA content [39]. In this study, the supplemented groups showed higher levels of acetic and butyric acids, with minimal changes in propionic acid. Nonetheless, no significant effects of treatment were observed for individual VFAs or total VFA levels. Therefore, the data do not strongly support the idea that dietary *L. acidophilus* altered cecal fermentation under the conditions of this experiment.

Appropriate probiotic supplementation in livestock and poultry diets has become a crucial strategy for improving animal health and production by balancing the intestinal microbiota. Probiotics are live microorganisms that, when consumed in adequate amounts, can provide health benefits to the animal. These beneficial microbes primarily promote the growth of beneficial bacteria, inhibit harmful bacteria, strengthen the intestinal barrier, and modulate the host immune system [40,41,42]. Studies show that adding *Bacillus subtilis* S62-9 to the diet can enhance the structure and diversity of the gut microbiota in broilers, increase beneficial bacteria, and reduce potential pathogenic bacteria [42]. Similarly, experiments by Qiu et al. demonstrated that supplementing broiler diets with a compound probiotic significantly raised *Lactobacillus* levels in the cecum, decreased *E. coli* counts, and lowered the *E. coli*-to-*Lactobacillus* ratio [43]. The results indicated that, in the cecum, the combined cultured counts of the two target bacterial groups in the T1 and T2 groups increased by 1.52% and 1.44%, respectively, compared to the control group (*p* < 0.05). This suggests that dietary *L. acidophilus* affected the culture-based counts of these bacteria in the cecum. Specifically, *Lactobacillus* counts rose by 8.36% and 9.03% (*p* < 0.05), while *E. coli* counts decreased by 4.98% and 5.83% (*p* < 0.05). These findings indicate a shift toward higher *Lactobacillus* and lower *E. coli* counts. Similar changes were seen in the ileum, but to a lesser extent, probably because the ileum is a site of digestion and absorption and is generally shorter, making it less suitable for extensive microbial colonization. Nevertheless, these changes were modest, and since the analysis focused on only two bacterial groups, the results should be regarded as targeted bacterial counts rather than broad shifts across the entire gut microbiota. Therefore, the probiotic effects on these bacterial counts in this section are relatively limited.

The structural features of the intestine, especially villus height, crypt depth, and their ratios, are key histological indicators for evaluating how probiotics affect intestinal health and digestion [44]. Among these, villus height usually reflects the absorptive surface area, while crypt depth is closely tied to epithelial renewal and metabolic demand. The ratio of these two measures offers a comprehensive view of the intestinal structure’s maturity and function [45]. Studies show that supplementing broiler diets with *B. subtilis* significantly increases villus height in the duodenum and ileum, along with improving the villus-to-crypt ratio in the ileum [46]. Similarly, in broilers infected with necrotic enteritis, adding butyric acid-producing bacteria effectively alleviates villus shortening and increases crypt depth [18]. In this study, particularly in the anterior intestine, such as the duodenum, adding *Lactobacillus* to the diet notably affected villus height, demonstrating a clear secondary effect. An additional 10 g/kg (T1) significantly increased villus height, whereas 15 g/kg (T2) did not further improve villus height. This suggests that an optimal level of *L. acidophilus* correlates with increased villus growth in the duodenum. However, because direct functional assessments, such as digestive enzyme activity or nutrient absorption tests, were not performed, these structural changes should be viewed as supportive evidence rather than definitive proof of enhanced absorption. Overall, villus height, crypt depth, and the V/C ratio in the jejunum and ileum remained consistent across treatments, with no significant differences; only numerical patterns were noted in some indicators. In summary, dietary *L. acidophilus* may influence intestinal structural features under specific conditions, but the functional relevance of these morphological changes warrants further investigation. Furthermore, although multiple endpoint categories were measured, the present study did not include multivariate analysis methods such as PCA, redundancy analysis, or correlation network analysis. As a result, the relationships among serum indices, targeted bacterial counts, intestinal morphology, nutrient utilization, and growth performance could not be fully understood.

## 5. Limitations

This study has several limitations to consider when interpreting the results. First, only two dietary inclusion levels of *L. acidophilus* were tested, limiting the ability to perform a full dose–response analysis or to determine the optimal supplementation level. Second, the analysis of intestinal bacteria was based on selective culture of *Lactobacillus* and *E. coli* rather than high-resolution sequencing-based profiling, meaning it reflects targeted bacterial counts rather than a complete microbiota profile. Third, a relatively simple GLM-based statistical approach was used for multiple endpoints, and no formal correction for multiple testing across all variables was applied. As a result, isolated significant responses should be interpreted with caution. Additionally, the study primarily relied on univariate analyses, and no multivariate methods (e.g., PCA or correlation-based analyses) were employed to explore relationships among performance, serum traits, bacterial counts, and intestinal morphology. Future studies incorporating multivariate integration would help clarify the biological relationships among these endpoints.

## 6. Conclusions

Under the conditions of this study, dietary *L. acidophilus* supplementation had limited effects on the overall growth performance of yellow-feathered broilers. A moderate inclusion level (10 g/kg) was associated with higher apparent nutrient digestibility of several nutrients between days 22 and 42, increased cultured *Lactobacillus counts*, decreased cultured *E. coli* counts in the intestine, and greater duodenal villus height. However, these responses were stage-specific and did not result in a significant overall improvement in growth performance by the end of the trial. Therefore, the current findings suggest that *L. acidophilus* may influence certain intestinal traits under specific production conditions, but the biological and practical significance of these effects should be interpreted with caution. Additionally, since this study lacked an economic evaluation or large-scale field validation, further research is necessary before making broader claims about the use of *L. acidophilus* in poultry production.

## Figures and Tables

**Table 1:** Feed ingredients and analyzed or calculated nutrient levels of experimental diets (on an air-dried basis).

Items	1 to 21 Days	22 to 42 Days	43 to 63 Days
Corn	62.97	60.53	63.36
Soybean meal 43%	25.80	20.80	17.70
Coen gluten meal 60%	5.00	5.00	5.00
Barley	0.00	6.00	6.00
Corn germ meal	1.60	2.70	1.70
Dicalcium phosphate	1.15	0.88	0.78
Talcum Powder	1.39	1.31	1.20
Cotton oil	0.60	1.40	2.30
NaCl	0.25	0.20	0.20
Lysine 98%	0.31	0.31	0.26
Solid methionine	0.19	0.17	0.00
Threonine	0.06	0.04	0.00
Premix	0.68	0.66	1.5
Total	100.00	100.00	100.00
Nutrient level			
DM (%)	89.74	89.44	88.45
OM (%)	95.51	96.34	94.89
CP (%)	19.38	16.55	16.45
ME (MJ/kg)	12.21	12.46	12.83
Ca	0.94	0.80	0.70
TP	0.60	0.57	0.52
Lys	1.15	1.04	0.93

Note: The NaCl values shown in Table 1 represent the amount of supplemental sodium chloride added to the diet. Total dietary sodium and chloride were derived from all dietary ingredients and additives, including choline chloride in the premix. The vitamin and mineral composition of the diet provided per kg was as follows: (1) 1 to 21 days of age: VA 12 000 IU; VD3 600 IU; VE 35 mg; VK3 2.5 mg; VB1 2.3 mg; VB2 5.0 mg; VB6 2.4 mg; VB12 16 mg; niacin 40 mg; calcium pantothenate 12 mg; biotin 0.11 mg; folic acid 1.0 mg; choline chloride 1300 mg; copper 7 mg; manganese 80 mg; zinc 85 mg; iodine 0.70 mg; selenium 0.15 mg; (2) 22 to 42 days of age: VA 7000 IU; VD3 500 IU; VE 25 mg; VK3 2.2 mg; VB1 2.2 mg; VB2 5.0 mg; VB6 2.0 mg; VB12 0.04 mg; niacin 35 mg; calcium pantothenate 10 mg; biotin 0.10 mg; folic acid 0.7 mg; choline chloride 1000 mg; copper 7 mg; manganese 60 mg; zinc 80 mg; iodine 0.60 mg; selenium 0.17 mg; (3) 43 to 63 days of age: VA 6000 IU; VD3 500 IU; VE 25 mg; VK3 1.7 mg; VB1 1.0 mg; VB2 4.0 mg; VB6 0.6 mg; VB12 0.04 mg; niacin 20 mg; copper 6.7 mg; manganese 70 mg; zinc 65 mg; iodine 0.60 mg; selenium 0.25 mg. DM, OM, and CP were analyzed values. ME, Ca, TP, and Lys were calculated values based on feed formulation and ingredient composition. DM, dry matter; OM, organic matter; CP, crude protein; ME, metabolizable energy; TP, total phosphorus; Lys, lysine.

**Table 2:** Effects of *L. acidophilus* supplementation on the production performance of yellow-feathered broilers (g/d).

Period	Items	Treatments			SEM	*p*-Value		
		Control	T1	T2		T	L	Q
1–21 d	ADFI	43.78	44.12	45.52	2.640	0.960	0.788	0.925
	ADG	24.77	24.80	25.26	0.260	0.721	0.530	0.623
	F: G ratio	1.77	1.78	1.80	0.019	0.802	0.587	0.715
22–42 d	ADFI	101.54	103.77	105.74	2.360	0.773	0.480	0.911
	ADG	48.69	50.83	50.59	0.745	0.468	0.271	0.601
	F: G ratio	2.09	2.05	2.10	0.029	0.785	0.959	0.497
43–63 d	ADFI	154.09	160.96	160.86	2.320	0.388	0.197	0.640
	ADG	57.36	59.58	61.84	0.963	0.167	0.068	0.699
	F: G ratio	2.69	2.71	2.61	0.038	0.593	0.504	0.446
1–63 d	ADFI	99.80	102.92	104.04	3.680	0.890	0.642	0.898
	ADG	43.60	45.07	45.90	2.240	0.918	0.681	0.990
	F: G ratio	2.29	2.29	2.27	0.020	0.875	0.623	0.899

T, overall treatment effect; L, linear effect; Q, quadratic effect.

**Table 3:** Effect of *L. acidophilus* supplementation on the apparent nutrient digestibility in yellow-feathered broilers (%).

Period	Items	Treatments			SEM	*p*-Value		
		Control	T1	T2		T	L	Q
1–21 d	DM	63.48	69.71	64.46	3.000	0.353	0.620	0.187
	OM	69.12	71.65	65.98	3.360	0.526	0.662	0.311
	EE	76.17	80.01	76.32	1.960	0.356	0.738	0.177
	CP	41.20	54.56	41.66	5.120	0.193	0.663	0.087
	GE	67.36	72.91	67.75	0.349	0.349	0.705	0.175
22–42 d	DM	63.15 ^b^	68.83 ^a^	65.17 ^b^	0.770	0.006	0.33	0.004
	OM	66.02 ^b^	70.84 ^a^	67.44 ^b^	0.808	0.014	0.091	0.009
	EE	61.16 ^b^	67.10 ^a^	63.06 ^b^	0.844	0.007	0.048	0.004
	CP	40.80 ^b^	57.38 ^a^	42.76 ^b^	2.900	0.013	0.241	0.006
	GE	68.30 ^b^	75.81 ^a^	72.72 ^a^	1.310	0.019	0.025	0.032
43–63 d	DM	67.32	66.17	64.99	1.350	0.516	0.278	0.821
	OM	68.77	69.28	67.52	0.811	0.351	0.425	0.231
	EE	65.79	64.74	63.46	1.500	0.576	0.327	0.795
	CP	35.60	37.05	31.74	1.690	0.150	0.247	0.105
	GE	76.38	76.01	75.08	0.628	0.384	0.222	0.550

Different superscript letters within the same row indicate significant differences among treatments (*p* < 0.05). Values were calculated based on nutrient intake and overall excreta output. Since fecal and urinary nitrogen are excreted together in poultry, CP values should be interpreted as apparent CP/N retention under the total excreta collection method rather than as ileal protein digestibility. Data were analyzed separately for each sampling period. The reported *p*-values indicate treatment, linear, and quadratic effects within the same period; sampling time and treatment × sampling time interactions are not reported in this table.

**Table 4:** Effect of *L. acidophilus* supplementation on serum protein metabolism and total bilirubin in yellow-feathered broilers.

Period	Items	Treatments			SEM	*p*-Value		
		Control	T1	T2		T	L	Q
1–21 d	TP (g/L)	27.18	27.66	27.54	1.380	0.968	0.834	0.892
	ALB (g/L)	11.46	12.08	11.70	0.542	0.723	0.661	0.509
	GLB (g/L)	15.72	15.44	15.84	0.921	0.952	0.974	0.759
	A/G	0.73	0.78	0.74	0.030	0.566	0.672	0.336
	BUN (mmol/L)	0.43 ^b^	0.59 ^a^	0.68 ^a^	0.056	0.023	0.007	0.955
	TBIL (mmol/L)	7.58	6.68	6.50	1.090	0.761	0.476	0.897
22–42 d	TP (g/L)	33.02	33.32	32.52	1.550	0.935	0.869	0.749
	ALB (g/L)	12.36	11.58	12.06	0.423	0.446	0.484	0.293
	GLB (g/L)	20.66	21.74	20.46	1.290	0.757	0.974	0.465
	A/G	0.60	0.54	0.59	0.031	0.309	0.610	0.154
	BUN (mmol/L)	0.59	0.60	0.66	0.030	0.239	0.160	0.340
	TBIL (mmol/L)	9.36	8.84	9.00	1.010	0.934	0.771	0.829
43–63 d	TP (g/L)	35.86	37.92	35.12	1.450	0.395	0.925	0.183
	ALB (g/L)	13.68	14.60	13.64	0.523	0.371	0.826	0.172
	GLB (g/L)	22.18	23.32	21.48	1.010	0.454	0.804	0.227
	A/G	0.62	0.63	0.64	0.020	0.880	0.633	0.893
	BUN (mmol/L)	0.39 ^b^	0.57 ^a^	0.41 ^b^	0.044	0.026	0.396	0.010
	TBIL (mmol/L)	9.28	8.50	7.38	1.000	0.431	0.220	0.704

Different superscript letters within the same row indicate significant differences among treatments (*p* < 0.05). Data were analyzed separately for each sampling period. The reported *p*-values indicate treatment, linear, and quadratic effects within the same period; sampling time and treatment × sampling time interactions are not reported in this table.

**Table 5:** Effect of *L. acidophilus* supplementation on serum glucose, lipid, and liver enzyme concentrations in yellow-feathered broilers.

Period	Items	Treatments			SEM	*p*-Value		
		Control	T1	T2		T	L	Q
1–21 d	TG (mmol/L)	0.63 ^b^	0.99 ^a^	0.78 ^b^	0.058	0.003	0.024	0.004
	TCH (mmol/L)	3.56	3.69	3.24	0.154	0.138	0.259	0.095
	LDL (mg/dL)	2.75	2.79	2.53	0.132	0.357	0.353	0.274
	HDL (mg/dL)	0.72	0.76	0.62	0.061	0.265	0.332	0.187
	Glu (mmol/L)	12.60	12.22	11.76	0.310	0.201	0.087	0.650
	AST (U/L)	176.40	252.80	201.00	24.700	0.124	0.296	0.075
	ALT (U/L)	3.60	3.20	3.80	0.283	0.344	0.831	0.156
22–42 d	TG (mmol/L)	1.23	1.27	1.21	0.159	0.966	0.976	0.797
	TCH (mmol/L)	3.91	3.54	3.78	0.118	0.119	0.266	0.076
	LDL (mg/dL)	3.06 ^a^	2.74 ^b^	2.89 ^a^	0.094	0.095	0.125	0.106
	HDL (mg/dL)	0.81	0.82	0.91	0.067	0.534	0.351	0.548
	Glu (mmol/L)	13.87	13.45	13.01	0.595	0.605	0.335	0.843
	AST (U/L)	249.00 ^a^	239.60 ^a^	219.20 ^b^	7.280	0.037	0.018	0.271
	ALT (U/L)	4.40	3.80	4.00	0.548	0.738	0.547	0.634
43–63 d	TG (mmol/L)	0.60 b	1.09 a	0.84 a	0.140	0.086	0.137	0.085
	TCH (mmol/L)	3.58	3.38	3.40	0.180	0.691	0.441	0.726
	LDL (mg/dL)	2.74	2.49	2.56	0.168	0.557	0.377	0.546
	HDL (mg/dL)	0.79	0.76	0.74	0.079	0.913	0.681	0.937
	Glu (mmol/L)	13.46	13.65	13.67	0.643	0.967	0.805	0.948
	AST (U/L)	203.20	219.80	200.40	13.400	0.560	0.952	0.292
	ALT (U/L)	1.20	1.60	1.60	0.231	0.397	0.206	0.652

Different superscript letters within the same row indicate significant differences among treatments (*p* < 0.05). Data were analyzed separately for each sampling period. The reported *p*-values indicate treatment, linear, and quadratic effects within the same period; sampling time and treatment × sampling time interactions are not reported in this table.

**Table 6:** Effects of dietary *L. acidophilus* supplementation on serum antioxidant status in yellow-feathered broilers.

Period	Items	Treatments			SEM	*p*-Value		
		Control	T1	T2		T	L	Q
22–42 d	IgA (ng/mL)	2.54	2.03	2.50	0.223	0.225	0.643	0.108
	IgG (ng/mL)	6.72	6.16	5.76	0.415	0.309	0.134	0.880
	IgM (ng/mL)	2.03	1.52	2.04	0.246	0.187	0.744	0.081
	CAT (U/mL)	0.20 ^b^	0.24 ^a^	0.32 ^a^	0.026	0.031	0.014	0.309
	GSH-Px (U/mL)	1899.72 ^ab^	1669.86 ^b^	2440.56 ^a^	183.000	0.032	0.129	0.024
	T-AOC (mmol/L)	0.64	0.74	0.70	0.038	0.227	0.235	0.204
	SOD (U/L)	399.45	420.64	412.01	18.200	0.715	0.555	0.582
	MDA (mmol/L)	2.76	2.96	2.76	0.304	0.857	0.918	0.594
43–63 d	IgA (ng/mL)	2.70 ^a^	2.09 ^b^	1.96 ^b^	0.176	0.027	0.010	0.618
	IgG (ng/mL)	7.20	7.81	7.24	0.543	0.667	0.833	0.402
	IgM (ng/mL)	2.14	2.66	2.22	0.205	0.185	0.540	0.095
	CAT (U/mL)	0.17 ^b^	0.31 ^a^	0.22 ^b^	0.019	<0.001	0.015	0.001
	GSH-Px (U/mL)	2846.20	2393.24	2609.58	157.000	0.166	0.193	0.156
	T-AOC (mmol/L)	0.60	0.64	0.69	0.030	0.127	0.054	0.557
	SOD (U/L)	469.69 ^a^	390.82 ^b^	390.58 ^b^	22.000	0.039	0.017	0.360
	MDA (mmol/L)	3.04	2.34	1.97	0.445	0.265	0.110	0.988

Different superscript letters within the same row indicate significant differences among treatments (*p* < 0.05). Data were analyzed separately for each sampling period. The reported *p*-values indicate treatment, linear, and quadratic effects within the same period; sampling time and treatment × sampling time interactions are not reported in this table.

**Table 7:** Effects of dietary *L. acidophilus* supplementation on carcass yield traits of yellow-feathered broilers (%).

Traits	Treatments			SEM	*p*-Value		
	Control	T1	T2		T	L	Q
Dressing percentage	92.09	92.34	91.89	0.294	0.319	0.604	0.158
Semi-eviscerated carcass yield	84.34 ^a^	83.07 ^ab^	82.56 ^b^	0.481	0.048	0.015	0.783
Eviscerated carcass yield	68.94	68.95	66.85	0.671	0.077	0.073	0.152
Breast muscle yield	21.33	22.75	21.67	0.573	0.300	0.595	0.147
Thigh muscle yield	24.13 ^a^	22.37 ^b^	23.56 ^a^	0.554	0.066	0.202	0.048

Different superscript letters within the same row indicate significant differences among treatments (*p* < 0.05).

**Table 8:** Effect of *L. acidophilus* supplementation on volatile fatty acids in the cecum of yellow-feathered broilers (mg/g).

Traits	Treatments			SEM	*p*-Value		
	Control	T1	T2		T	L	Q
Acetic acid	18.30	18.68	22.56	2.050	0.333	0.237	0.373
Propionic acid	4.83	4.60	5.21	0.247	0.279	0.448	0.163
Isobutyric acid	0.32	0.32	0.38	0.047	0.622	0.455	0.555
Butyric acid	5.24	5.93	7.99	0.917	0.169	0.098	0.357
Valeric acid	0.35	0.35	0.43	0.058	0.559	0.454	0.453
Pentanoic acid	0.41	0.49	0.59	0.072	0.273	0.133	0.656
TVFA	29.44	30.36	37.16	3.040	0.227	0.159	0.308

**Table 9:** Effect of *L. acidophilus* supplementation on intestinal morphology of yellow-feathered broilers (μm).

Organ	Items	Treatments			SEM	*p*-Value		
		Control	T1	T2		T	L	Q
Duodenum	Villus height	603.75 ^b^	810.67 ^a^	587.19 ^b^	37.400	0.001	0.571	<0.001
	Crypt depth	69.59	96.40	80.28	7.770	0.079	0.187	0.060
	(V/C)	8.74	8.82	7.62	0.715	0.434	0.355	0.370
Jejunum	Villus height	539.61	529.85	457.29	52.000	0.490	0.330	0.497
	Crypt depth	107.35	72.90	80.19	14.400	0.237	0.146	0.377
	(V/C)	5.43	7.30	6.18	0.639	0.150	0.260	0.106
Ileum	Villus height	372.12	368.88	452.05	47.400	0.395	0.319	0.354
	Crypt depth	56.07	59.30	57.57	4.350	0.873	0.748	0.688
	(V/C)	6.65	6.32	7.85	0.582	0.184	0.255	0.143

Different superscript letters within the same row indicate significant differences among treatments (*p* < 0.05).

## Data Availability

The original contributions presented in this study are included in the article. Further inquiries can be directed to the corresponding author.

## Abbreviations

The following abbreviations are used in this manuscript:

ADFI	Average daily feed intake
ADG	Average daily gain
F: G	Feed-to-gain ratio
TP	Total protein
ALB	Albumin
GLB	Globulin
BUN	Blood urea nitrogen
TBIL	Total bilirubin
TG	Triglycerides
TCH	Total cholesterol
LDL	Low-density lipoprotein
HDL	High-density lipoprotein
Glu	Glucose
AST	Aspartate aminotransferase
ALT	Alanine aminotransferase
IgA	Immunoglobulin A
IgG	Immunoglobulin G
IgM	Immunoglobulin M
CAT	Catalase
GSH-Px	Glutathione peroxidase
T-AOC	Total antioxidant capacity
SOD	Superoxide dismutase
MDA	Malondialdehyde
VFA	Volatile fatty acid
TVFAs	Total volatile fatty acids
VH	Villus height
CD	Crypt depth
V/C	Villus height/crypt depth ratio
